# Conformational Aspects of the *O*-acetylation of *C-*tetra(phenyl)calixpyrogallol[4]arene

**DOI:** 10.3390/molecules23051225

**Published:** 2018-05-20

**Authors:** José Luis Casas-Hinestroza, Mauricio Maldonado

**Affiliations:** Departamento de Química, Facultad de Ciencias, Universidad Nacional de Colombia-sede Bogotá, Carrera 30 # 45-03, Bogotá 111321, Columbia; jlcasash@unal.edu.co

**Keywords:** macrocycles, pyrogallolarenes, conformers, acetylation

## Abstract

Reaction between pyrogallol and benzaldehyde results in a conformational mixture of *C-*tetra(phenyl)pyrogallol[4]arene (crown and chair). The conformer mixture was separated using crystallization procedures and the structures were determined using FTIR, ^1^H-NMR, and ^13^C-NMR. *O*-acetylation of *C-*tetra(phenyl)pyrogallol[4]arene (chair) with acetic anhydride, in pyridine results in the formation of dodecaacetyl-tetra(phenyl)pyrogallol[4]arene. The structure was determined using ^1^H-NMR and ^13^C-NMR finding that the product maintains the conformation of the starting conformer. On the other hand, the *O*-acetylation reaction of *C-*tetra(phenyl)pirogallol[4]arene (crown) under same conditions proceeded efficiently, and its structure was determined using ^1^H-NMR and ^13^C-NMR. Dynamic ^1^H-NMR of acetylated pyrogallolarene was studied by means of variable temperature in DMSO-*d*_6_ solution, and it revealed that two conformers are formed in the solution. Boat conformations for acetylated pyrogallolarene showed a slow interconversion at room temperature.

## 1. Introduction

Pyrogallol[4]arenes are cavity-shaped macrocylces which can be synthesized from pyrogallol and aromatic [1,2,3] or aliphatic aldhydes [4]. They have attracted much interest in the field of supramolecular chemistry due to the ease of forming capsules [5] with different types of reagents such as water [6], ammonium salts [7], copper [8], gallium [9], and fluorophores including 4-[3-(9-anthryl)propyl]-*N,N*-dimethylaniline(ADMA). Pyrogallol[4]arenes derivatives exhibit activities such as serving as chemosensors for visualization of metallic cations [10,11,12], anions [13], and neutral molecules such as coumarin [14]. In addition, they can be used as vesicle [15] and synthetic ion channels [16].

Functionalizing of pyrogallol[4]arenes can be done from the starting materials, varying the nature of the substituent group on the aldehyde which facilitates modification of the lower rim of the macrocyclic system [17]. On the upper rim, the obvious sites for chemical modification are the hydroxyl groups since they can be modified with phosphoryl groups [11], carboxymethyl groups [12], acetyl hydrazine groups [18], and imidazolium groups [13], among others [17]. An interesting reaction is the acetylation of the hydroxyl groups. During the last decade, reaction studies of *O*-acetylation of pyrogallolarenes with acetic anhydride in pyridine showed that the main product of the reaction is the polyacetylated macrocycle in the chair conformation [10,19,20] and showed that the reaction of the hydroxyl groups does not affects the conformation of the macrocyclic system if the conformation of the starting pyrogallolarene is fixed in the chair conformation. However, in these investigation there is not reference to the result of the reaction with the crown conformer.

As a follow-up to our studies on the reactivity of polihydroxylated macrocyclics [21,22,23,24] and in order to establish what happens with the crown conformer, in the present study we show the reaction between *C-*tetra(phenyl)pyrogallol[4]arene (fixed in crown or chair conformation) and acetic anhydride; in pyridine to generate polyacetyl-tetra(phenyl)pyrogallol[4]arene. The products were analyzed using ^1^H and ^13^C-NMR and FTIR spectroscopy, elemental analysis, mass spectrometry, and dynamic ^1^H-NMR spectroscopy. From the results, it is evident the important role played by the acetyl group, since it modulates the flexibility of the pyrogallolarene scaffold and the conformational properties of the resulting product.

## 2. Results and Discussion

**Scheme 1:**
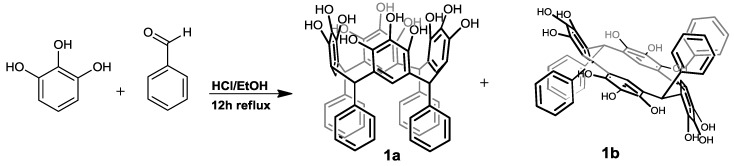
Synthesis of *C-*tetra(phenyl)pyrogallol[4]arene.

The synthesis of *C-*tetra(phenyl)pyrogallol[4]arene was accomplished by refluxing a mixture of equal amounts of pyrogallol and benzaldehyde in the presence of concentrated HCl in ethyl alcohol at 85 °C (Scheme 1). This derivative was characterized using spectral techniques, including FTIR and ^1^H-NMR (see Experimental section).

**Figure 1:**
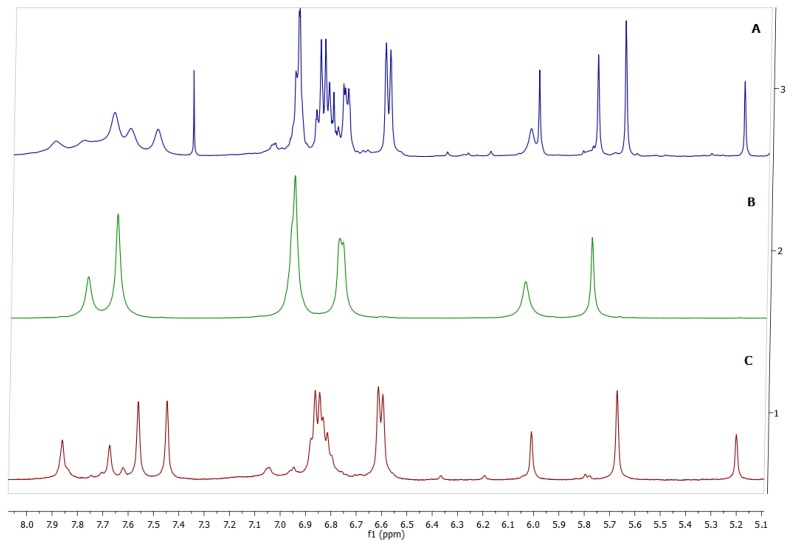
(**A**) ^1^H-NMR spectrum of conformational mixture: crown conformation **1a** and chair conformation **1b**; (**B**) ^1^H-NMR spectrum of pure conformer **1a**; and (**C**) ^1^H-NMR spectrum of pure conformer **1b**.

Careful analysis of ^1^H-NMR spectra of the crude showed two products corresponding to the conformational mixture: crown (**1a**) and chair (**1b**). These isomers possess well-resolved signals in the aliphatic region of their ^1^H-NMR spectra (Figure 1), allowing easy assignment of the two conformers and inclusion of their molar ratios in the crude product (43/57 crown/chair). After separation by means recrystallizations with several solvents (see experimental), the two products were isolated. The ^1^H-NMR spectra of the first product showed two single peaks, at 7.77 and 7.65 ppm corresponding to two classes of hydroxyl groups, the first signal corresponding to the central hydroxyl group in the pyrogallol ring and the second signal to two hydroxyl group in the *ortho* position. Additionally, the ^1^H-NMR spectrum displayed characteristic signals of phenyl substiuent in 6.95 and 6.77 ppm, a methylene bridge fragment between the aromatic rings (5.78 ppm) and one signal for the aromatic hydrogen of a pentasubstituted pyrogallol unit (6.04 ppm). In this way, the NMR spectroscopy analysis showed the highly symmetrical structure and all the patterns were consistent with the expected crown conformer **1a**, to our knowledge, this is the first time that this conformer has been isolated in its pure form. On the other hand, the spectrum of the second product displayed four different hydroxyl moieties, with two signals at 7.86 and 7.67 ppm assigned to the central hydroxyl group in the pyrogallol ring, and two signals at 7.56 and 7.45 ppm corresponding to hydroxyl groups in the *ortho* position attached to pyrogallol residues in the macrocyclic system. Careful analysis of all the patterns confirmed the structure of the chair conformer **1b** (Figure 1). This conformer has been previously synthesized by other authors, and their spectroscopic data agreed with those reported by us [1]. The carbon signals for **1a** and **1b** in ^13^C-NMR were unambiguously assigned through 1D- and 2D-NMR experiments, including the HMQC and HMBC spectra. The signals for carbons linked to hydrogen were assigned based on the observed correlations in the HMQC spectrum. Signals that did not show any correlation in the HMQC spectrum were those that were not linked to protons.

**Figure 2:**
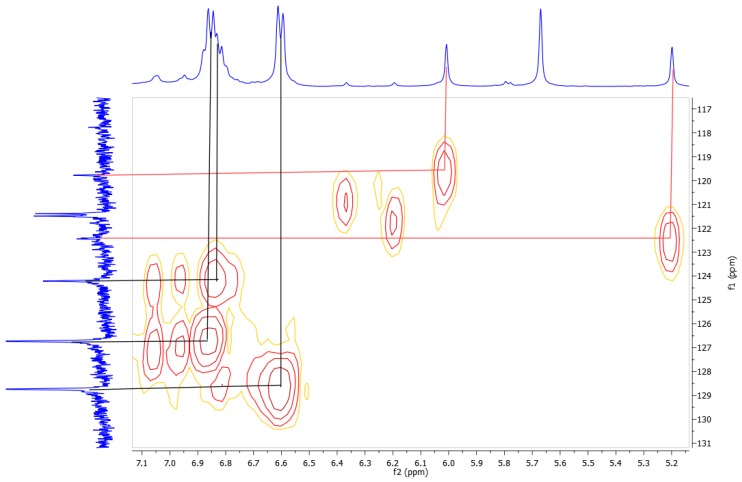
HMQC spectrum of conformer **1b**.

An interesting aspect of the ^1^H-NMR spectrum of **1b** is the position of the signals of the aromatic resonance of the pyrogallol residue which appears downfield at 6.01 and 5.20 ppm. As mentioned above, the assignment of these protons was done with the HMQC spectrum. In the HMQC spectrum, the signal at 122.4 ppm shows a correlation with the aromatic protons (6.01 ppm) and the signal at 119.8 ppm has a correlation with the signals at 5.20 and confirm that these signals correspond to the aromatic system (Figure 2).

**Scheme 2:**
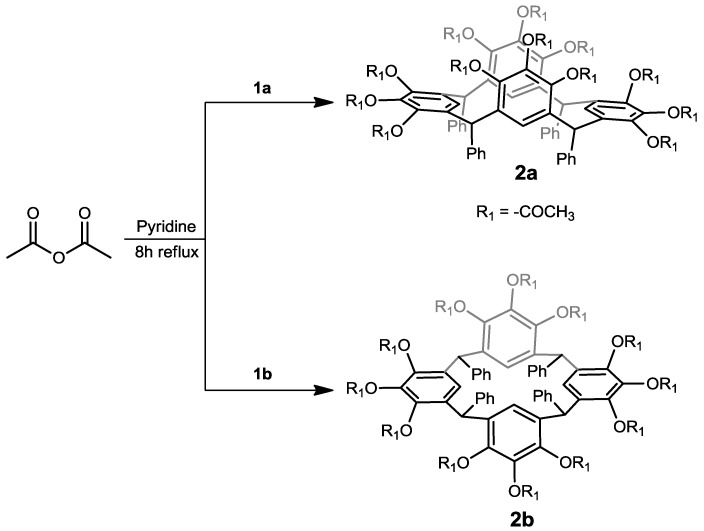
Synthesis of *C-*tetraphenylpyrogallol[4]arene acetylated.

Pyrogallolarenes **1a** and **1b** were subjected to an acetylation reaction using a mixture of acetic anhydride and pyridine producing dodecaacetyl-tetra(phenyl)pyrogallol[4]arene (conformers **2a** and **2b** respectively) and the products were purified by means of recrystallization (Scheme 2). These derivatives were characterized using spectral techniques, including FTIR, ^1^H-NMR, ^13^C-NMR and mass spectrometry (see experimental section). Compound **2b** had been previously synthesized by other authors [19], and the spectroscopic data agreed with those reported by us. The ^1^H-NMR spectrum of **2b** displayed three characteristic signals in the aliphatic region due to acetyl groups (2.12 and 2.36 ppm), and a methylene bridge fragment between the aromatic rings (5.30 ppm). In the aromatic region, hydrogens of the pentasubstituted pyrogallol unit appearing at 6.31 and 6.07 ppm and signals of the phenyl groups were observed at 6.77 and 7.04 ppm. The ^13^C-NMR spectrum in DMSO-*d*_6_ displayed characteristic signals of acetyl groups (20.8 and 19.6 ppm) and the signal at 44.2 ppm confirmed the presence of a methylene bridge fragment between the aromatic rings. In addition to the signals of the aromatic region (12 signals) C=O of carbonyl groups were confirmed at 171.6 ppm and 166.4 ppm. These spectroscopic data could indicate that **2b** has a chair conformation which would be consistent with previous findings in recent papers.

**Figure 3:**
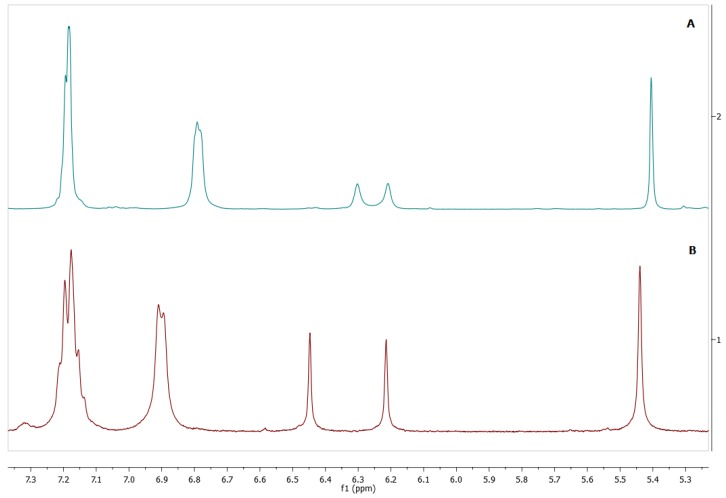
Comparison of the fragments of ^1^H-NMR spectra of the conformer **2a** (**A**) and **2b** (**B**).

Compound **2a** was obtained as a white solid. The FTIR spectrum is in agreement with the organic functionalities present in the structure of the compound, since it reveals ester group stretches at 1778 cm^−1^ (C=O) and 1162 cm^−1^ (C-O), whereas bands of the alkyl substituent and the aromatic ring are also observed. The ^1^H-NMR spectrum displayed characteristic signals at room temperature of acetyl groups at 2.08 and 2.17 ppm, and of the methylene bridge fragment between the aromatic rings at 5.40 ppm. In the aromatic region, the ^1^H-NMR spectrum displayed two signals for the proton in the pentasubstituted pyrogallol unit at 6.21 and 6.30 ppm and the phenyl substituent in the lower rim displayed two signals at 6.79 and 7.18 ppm. According to these spectroscopic data, as expected, the NMR spectra of the two conformers were similar as can be seen in Figure 3.

**Figure 4:**
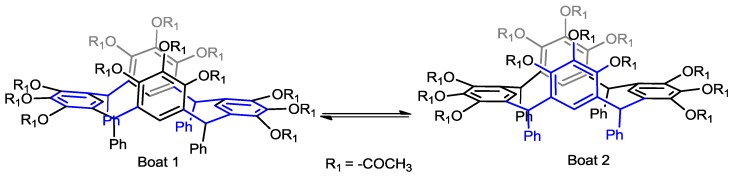

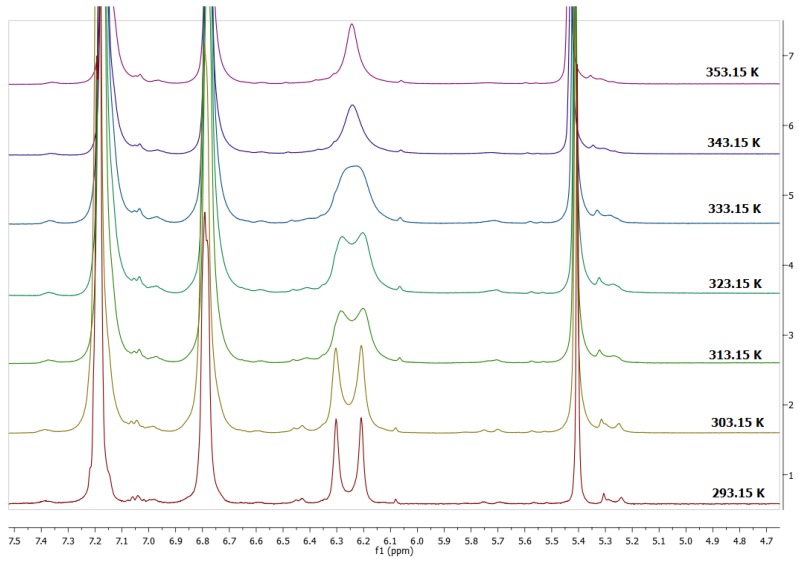
Coalescence of the peaks in the aromatic zone for conformer **2a**.

In order to establish the conformational behavior of **2a** and **2b** we decided to carry out dynamic ^1^H-NMR study of acetylated pyrogallolarenes. Initially they were studied for conformer **2b** by means of variable temperature in DMSO-*d_6_* solution but the signals in the spectrum did not change and revealed that only one conformer is present in the solution. This result confirmed that the conformation of acetylated pyrogallolarene **2b** is chair. In contrast to this result, the dynamic ^1^H-NMR study of **2a** did show the presence of two conformers (Figure 4).

The ^1^H-NMR spectra of compound **2a** was consistent with the presence of two conformers, boat 1 (B1) and boat 2 (B2). Thus, at 293 K the spectrum of compound **2a** in the aromatic region exhibited two signals for resonances of aromatic protons of pyrogallol residue on the lower rim at 6.30 ppm (hydrogen in the flattened ring) and 6.20 ppm (hydrogen in the opposite ring). Other areas of the spectrum are consistent with this conformational assignment, and variable temperature effects were also observed (Figure 4). The temperature of coalescence was found to be 343 K.

The assignment of the signals for each conformer and the information of the aromatic protons in DMSO-*d*_6_ provided data for the calculation of the conformer interconversion. The rate constants, *Kc*, of dodecaacetyl-tetra(phenyl)pyrogallol[4]arene interconvertion were calculated using the peak separation which was obtained from spectra with the signals in the aromatic region at different temperatures. Based on these data and using the Eyring method, the free energy associated with the process of conformational exchange (ΔG_298_) was estimated to be 70.45 KJ mol^−1^. This value is slightly higher than for other esters of polyhidroxylated macrocycles which range from 55 to 65 kJmol^−1^ [25,26]. These differences in the interconversion could be related to the nature of the solvent, which governs the ability to form interactions between aryl groups in the acetylated pyrogallol[4]arene system. Therefore, the bulky phenyl substituents on the lower rim of the macrocycle are free and completely out of the pyrogallol[4]arene cavity, and consequently they can form additional interactions with the solvent. This system is less dynamic than others and all the groups are involved between the bonded and the free state, therefore the energy barrier is higher. The high value of the conformational exchange (ΔG_298_) suggests greater interactions in boat conformations than in crown conformations. These interactions could be caused by the absence of a hydrogen bond on the upper rim, solvent-solvent interactions, and van der Waals interactions between the appending substituents in the lower rim of pyrogallol[4]arene.

## 3. Materials and Methods

### 3.1. General Experimental Information

IR spectra were recorded on a ThermoFisher Scientific Nicolet iS10 FTIR spectrometer with a monolithic Diamond ATR accessory and absorption in cm^−1^ (Thermo Scientific, Waltham, MA, USA). ^1^H and ^13^C-NMR spectra were recorded at 400 MHz on a Bruker Avance 400 instrument. Molar mass was determined with MALDI–TOF spectrometer (Bruker Daltonics, Bremen, Germany) using 4-nitroaniline as a matrix for the desorption/ionization process. RP–HPLC analyses were performed on Chomolith^®^ C18 column (Merck, Kenilworth, NJ, USA, 50 mm) using an Agilent 1200 Liquid Chromatograph (Agilent, Omaha, NE, USA). Chemical shifts are reported in ppm, using the solvent residual signal. Melting points were measured on a Stuart apparatus (Cole-Parmer, Stafford, UK) and are not corrected. The elemental analysis for carbon and hydrogen was carried out using a Thermo Flash 2000 elemental analyzer (Thermo Scientific, Waltham, MA, USA). 

### 3.2. Synthesis of C-tetra(phenyl)pyrogallol[4]arene

A Pyrogallol solution (5 mmol) in 20 mL of ethanol was addied dropwise at 0.4 mL of concentrated chlorine acid (37%), the mixture was stirred at 0 °C for 5 min and then 0.5 mL of benzaldehyde (5 mmol) was added dropwise. After 10 min the mixture was heated to 70–80 °C for 12 h, and the solid precipitate was filtered and washed with a mixture of water and ethanol (1:1) producing a pink solid (569 mg) in 56% yield, which was characterized by means IR, ^1^H-NMR and ^13^C-NMR. The separation of boat and chair conformer was carried out by using solvent-extraction technique with mixtures of solvents such as ethyl acetate, water and ethanol. The crude mixture (200 mg) was stirred in 10 mL of a mixture of solvents (ethyl acetate, ethanol, and water, at a ratio of 2:1:1, respectively) for 10 min, and the suspended solid was separated by filtration and dried, whereupon it was determined to be in the chair conformer configuration. The mixture of solvents was removed by evaporation, resulting in a second solid that exhibited the boat conformer. 

*C*-tetra(*phenyl*)pyrogallol[4]arene (*crown*) (**1a**) m.p. > 350 °C IR (ATR/cm^−1^): 3300–3600 (broad), 1630, 1500, 1464, 1368, 1282, 1247, 1209, 1064, 1015, 163, 699, 566, 416 cm^−1^; ^1^H-NMR (400 MHz, DMSO-*d*_6_): δ 7.77 (s, 4H, OH); 7.65 (s, 8H, OH); 6.95–6.96 (m, 12H, Ar); 6.76–6.77 (m, 8H, Ar); 6.04 (s, 4H, Ar); 5.78 (s, 4H, CH). ^13^C-NMR (100 MHz, DMSO-*d_6_*): δ 145.4; 142.0; 131.8; 128.6; 127.0; 124.5; 121.3. Calcd. For C_52_H_40_O_12_ (%) C 72.89; H 4.72; O 22.41. Found: C 73.85; H 4.67; O 21.49.

*C*-tetra(*phenyl*)pyrogallol[4]arene (*chair*) (**1b**) m.p. > 350 °C IR (ATR/cm^−1^): 3300–3600 (broad), 1630, 1500, 1464, 1368, 1282, 1247, 1209, 1064, 1015, 163, 699, 566, 416 cm^−1^; ^1^H-NMR (400 MHz, DMSO-*d*_6_): δ 7.86 (s, 2H, OH); 7.67 (s, 2H, OH); 7.56 (s, 4H, OH); 7.45 (s, 4H, OH), 6.84 (s, 12H, Ar); 6.60 (d, *J* = 8 Hz, 8H, Ar); 6.01 (s, 2H, Ar); 5.67 (s, 4H, CH); 5.20 (s, 2H, Ar). ^13^C-NMR (100 MHz, DMSO-*d_6_*): δ 143.8; 141.9; 141.6; 131.8; 131.3; 128.7; 126.7; 124.2; 122.4; 121.5; 121.4; 119.8; 42.6. Calcd. For C_52_H_40_O_12_ (%) C 72.89; H 4.72; O 22.41. Found: C 73.52; H 4.21; O 21.91.

### 3.3. Acetylation of C-tetra(phenyl)pyrogallol[4]arene

A mixture of tetra(phenyl)pyrogallol[4]arene (0.500 mmol) crown (**1a**) or chair (**1b**) conformers, acetic anhydride (16 mL) and pyridine (0.300 mL) was refluxed for 8h. The resultant precipitate was collected by filtration and washed with a mixture of water and ethanol (1:1) to give a white solid (73%) chair conformer and 62% boat conformer. 

Dodecaacetyl-tetra(*phenyl)*pyrogallol[4]arene (*boat*) (**2a**) m.p.: 302–304 °C. IR (ATR/cm^−1^): 3066, 2937, 1815, 1778, 1476, 1440, 1368, 1310, 1197, 1162, 1123, 1069, 1031, 952, 894, 872, 707, 676, 634 cm^−1^; ^1^H-NMR (400 MHz, DMSO-*d*_6_): δ 7.18-7.19 (m, 12H, Ar); 6.78-6.79 (m, 8H, Ar); 6.30 (s, 2H, Ar); 6.21 (s, 2H, Ar); 5.41 (s, 4H, CH); 2.17 (s, 12H, CH_3_); 2.08 (d, 24H, -CH_3_). ^13^C-NMR (100 MHz, DMSO-*d_6_*): δ 167.1; 166.9; 141.2; 136.1; 139.4; 136.1; 132.3; 131.9; 128.5; 128.1; 127.0; 44.0; 19.8; 19.6. MALDI-MS 1361.159 [M + H]^+^. Anal. Calcd. For C_76_H_64_O_24_: C, 67.05; H, 4.74; O, 28.21. Found: C, 65.65; H, 4.87; O, 29.48.

Dodecaacetyl-tetra(*phenyl*)pyrogallol[4]arene (*chair*) (2b) m.p.: 330–332 °C. IR (ART/cm^−1^): 3066, 2937, 1815, 1778, 1476, 1440, 1368, 1310, 1197, 1162, 1123, 1069, 1031, 952, 894, 872, 707, 676, 634 cm^−1^; ^1^H-NMR (400 MHz, DMSO-*d*_6_): δ 7.01–7.07 (m, 12H, Ar); 6.75–6.77(m, 8H, Ar); 6.31 (s, 2H, Ar); 6.07 (s, 2H, Ar); 5.30 (s, 2H, Ar); 2.08-2.16 (m, 36H, CH_3_). ^13^C-NMR (100 MHz, DMSO-*d*_6_): δ 167.3; 166.7; 166.5; 166.3; 140.2; 139.8; 137.8; 136.0; 135.4; 132.8; 132.6; 128.6; 128.2; 127.5; 126.3; 125.0; 44,4; 20.8; 19.6; 19.5; 19.4. MALDI-MS 1361.159 [M + H]^+^. Anal. Calcd. For C_76_H_64_O_24_: C, 67.05; H, 4.74; O, 28.21. Found: C, 65.46; H, 4.93; O, 29.57.

### 3.4. Dynamic NMR Experiments

The dynamic ^1^H-NMR experiment was performed using a sample of **2a**, and we observed a set of 1D spectra within temperature range of 293–353 K. For the NMR measurements, the pyrogallol[4]arene concentrations was 0.035 M in DMSO-*d*_6_.

## 4. Conclusions

Synthesis of *C-*tetra(phenyl)pyrogallol[4]arene produced a conformational mixture which had a ratio of 57% chair conformer to 43% crown conformer which was efficiently separated. The *O*-acetylation reaction of *C-*tetra(phenyl)pyrogallol[4]arene, fixed in chair or crown, demonstrated that the chair conformer retains the conformation and the crown conformer changes to the *boat* conformer. Dodecaacetyl-tetra(phenyl)pyrogallol[4]arene conformers were characterized using FTIR, ^1^H-NMR, and ^13^C-NMR spectral studies. Variable temperature ^1^H-NMR experiments confirmed that this conformational change increases and then drastically decreases the separations of the attached acetyl groups thus altering their interactions as well as producing a significant difference in the nature of the substituent on the lower rim of pyrogallol[4]arene. It was possible to demonstrate the conformational change through thermodynamic studies of the rotational barrier. The barrier to the conformational equilibrium of the macrocyclic system was studied and compared with analog macrocyclic systems in DMSO-*d*_6_ and the nature of phenyl substituent was found to be the main factor affecting the dynamic process observed in this acetylated macrocyclic. 

## Supplementary Materials

The Supplementary Materials are available online.

## References

[1] Maerz A.K., Thomas H.M., Power N.P., Deakyne C.A., Atwood J.L. (2010). Dimeric nanocapsule induces conformational change. Chem. Commun..

[2] Funck M., Guest D.P., Cave G.W.V. (2010). Microwave-assisted synthesis of resorcin[4]arene and pyrogallol[4]arene macrocycles. Tetrahedron. Lett..

[3] Kass J.P., Zambrano C.H., Zeller M., Hunter A.D., Dueno E.E. (2006). 2,8,14,20-Tetraphenylpyrogallol[4]arene dimethylformamide octasolvate. Acta Crystallogr. Sect. E Struct. Rep. Online.

[4] Manzano S., Zambrano C.H., Mendez M.A., Dueno E.E., Cazar R.A., Torres F.J. (2014). A theoretical study of the conformational preference of alkyl- and aryl-substituted pyrogallol[4]arenes and evidence of the accumulation of negative electrostatic potential within the cavity of their rccc conformers. Mol. Simul..

[5] Antesberger J., Cave G.W.V., Ferrarelli M.C., Heaven M.W., Raston C.L., Atwood J.L. (2005). Solvent-free, direct synthesis of supramolecular nano-capsules. Chem. Commun..

[6] Macgillivray L.R., Atwood J.L. (1997). A chiral spherical molecular assembly held together by 60 hydrogen bonds. Nature.

[7] Shivanyuk A., Friese J.C., Döring S., Rebek J. (2003). Solvent-Stabilized Molecular Capsules. J. Org. Chem..

[8] Jin P., Dalgarno S.J., Warren J.E., Teat J., Atwood J.L. (2009). Enhanced control over metal composition in mixed Ga/Zn and Ga/Cu coordinated pyrogallol[4]arene nanocapsules. Chem. Commun..

[9] Jin P., Dalgarno S.J., Barnes C., Teat S.J., Atwood J.L. (2008). Ion Transport to the Interior of Metal—Organic Pyrogallol[4]arene Nanocapsules. J. Am. Chem. Soc..

[10] Waidely E., Pumilia C., Malagon A., Vargas E.F., Li S., Leblanc R.M. (2015). Host-guest complexation of a pyrogallol[4]arene derivative at the air-water interface. Langmuir.

[11] Nikolelis D.P., Raftopoulou G. (2009). Development of an electrochemical chemosensor for the rapid detection of zinc based on air stable lipid films with incorporated calix[4]arene phosphoryl receptor. Int. J. Environ. Anal. Chem..

[12] Pod’yachev S.N., Mustafina A.R., Koppehele A.H., Grüner M., Habicher W.D., Buzykin B.I., Konovalov A.I. (2004). Synthesis of per-*O*-(carboxymethyl )calix[4]pyrogallols and their complexation with some alkaline metal and lanthanide ions. Russ. Chem. Bull..

[13] Kim S.K., Kang B., Koh H.S., Yoon Y.J., Jung S.J., Jeong B., Lee K., Yoon J. (2004). A New Imidazolium Cavitand for the Recognition of Dicarboxylates. Org. Lett..

[14] Chandrasekaran S., Yousuf S., Enoch I.V.M.V., Chandrasekaran S., Yousuf S., Enoch I.V.M.V. (2014). C-hexylpyrogallol[4]arene and β-cyclodextrin as directors of the mode of binding of Coumarin 153 with DNA. Supramol. Chem..

[15] Tanaka Y., Miyachi M., Kobuke Y. (1999). Selective vesicle formation from calixarenes by self-assembly. Angew. Chem. Int. Ed..

[16] Gokel G.W., Negin S. (2013). Synthetic Ion Channels: From Pores to Biological Applications. Acc. Chem. Res..

[17] Barrett E.S., Dale T.J., Rebek J. (2007). Synthesis and assembly of monofunctionalized pyrogallolarene capsules monitored by fluorescence resonance energy transfer. Chem. Commun..

[18] Podyachev S.N., Syakaev V.V., Sudakova S.N., Shagidullin R.R., Osyanina D.V., Avvakumova L.V., Buzykin B.I., Latypov S.K., Bauer I., Habicher W.D. (2007). Synthesis of new calix[4]arenes functionalizated by acetylhydrazide Groups. J. Incl. Phenom. Macrocycl. Chem..

[19] Han J., Song X., Liu L., Yan C. (2007). Synthesis, crystal structure and configuration of acetylated aryl Pyrogallol[4]arenes. J. Incl. Phenom. Macrocycl. Chem..

[20] Yan C., Chen W., Chen J., Jiang T., Yao Y. (2007). Microwave irradiation assisted synthesis, alkylation reaction, and configuration analysis of aryl pyrogallol[4]arenes. Tetrahedron.

[21] Sanabria E., Esteso M.Á., Vargas E., Maldonado M. (2018). Experimental comparative study of solvent effects on the structure of two sulfonated resorcinarenes. J. Mol. Liq..

[22] Maldonado M., Sanabria E., Batanero B., Esteso M.Á. (2017). Apparent molal volume and viscosity values for a new synthesized diazoted resorcin[4]arene in DMSO at several temperatures. J. Mol. Liq..

[23] Castillo-Aguirre A., Rivera-Monroy Z., Maldonado M. (2017). Selective *O*-alkylation of the crown conformer of tetra(4-hydroxyphenyl)calix[4]resorcinarene to the corresponding tetraalkyl ether. Molecules.

[24] Sanabria E., Esteso M.A., Prez-Redondo A., Vargas E., Maldonado M. (2015). Synthesis and characterization of two sulfonated resorcinarenes: A new example of a linear array of sodium centers and macrocycles. Molecules.

[25] Velásquez-Silva A., Cortés B., Rivera-Monroy Z.J., Pérez-Redondo A., Maldonado M. (2017). Crystal structure and dynamic NMR studies of octaacetyl-tetra(propyl)calix[4] resorcinarene. J. Mol. Struct..

[26] Abis L., Dalcanale E., Du Vosel A., Spera S. (1990). Nuclear Magnetic Resonance Elucidation of Ring-Inversion Processes in Macrocyclic Octaols. J. Chem. Soc. Perkin Trans. Phys. Org. Chem..

