# Supplementary material for: Conformational Aspects of the O-acetylation of C-tetra(phenyl)calixpyrogallol[4]arene
Source: Molecules. 2018 May 20;23(5):1225. doi: 10.3390/molecules23051225 (PMC6100403; doi:10.3390/molecules23051225)
Supplement: Supplementary file 1 [file molecules-23-01225-s001.pdf]

Conformational aspects of the *O*-acetylation of *C*-tetraphenylcalixpyrogallol[4]arene

José Luis Casas, Mauricio Maldonado

Table of contents:

Scheme S1. Synthesis of *C*-tetra(phenyl)pyrogallol[4]arene

Figure S1. ^{1}H-NMR spectrum - DMSO-d_6 conformational mix (**1a** and **1b)**

Figure S2. Tetra(phenyl)pyrogallol[4]arene (*crown*)(**1a**)

Figure S3. IR Spectrum of compound **1a**

Figure S4. ^{1}H-NMR spectrum DMSO-d_6 of boat conformer**(1a)**

Figure S5. Spectrum ^{13}C-NMR -DMSO-d_6 boat conformer**(1a)**

Figure S6. Tetra(phenyl)pyrogallo[4]larene (*chair*)(**1b**)

Figure S7. IR Spectrum of compound **1b**

Figure S8. ^{1}H-NMR of chair conformer(**1b**)

Figure S9. ^{13}C-NMR of chair conformer(**1b**)

Scheme S2. Synthesis of *C*-tetraphenylpyrogallol[4]arene acetylated (**2a** and **2b**)

Figure S10. Dodecaacethyl-tetra(phenyl)pyrogallol[4]arene (*Boat*)(**2a**)

Figure S11. IR spectrum of compound **2a**

Figure S12. ^{1}H-NMR spectrum boat isomer (**2a)**

Figure S13. ^{13}C-NMR boat conformer(**2a**)

Figure S14. Dodecaacethyl-tetra(phenyl)pyrogallol[4]arene (*chair*) (**2b**)

Figure S15. IR of compound **2b**

Figure S16. ^{1}H-NMR spectrum of chair isomer(**2b**)

Figure S17. ^{13}C–NMR spectrum of chair isomer(**2b**)

Synthesis of tetraphenylpyrogallol[4]arene

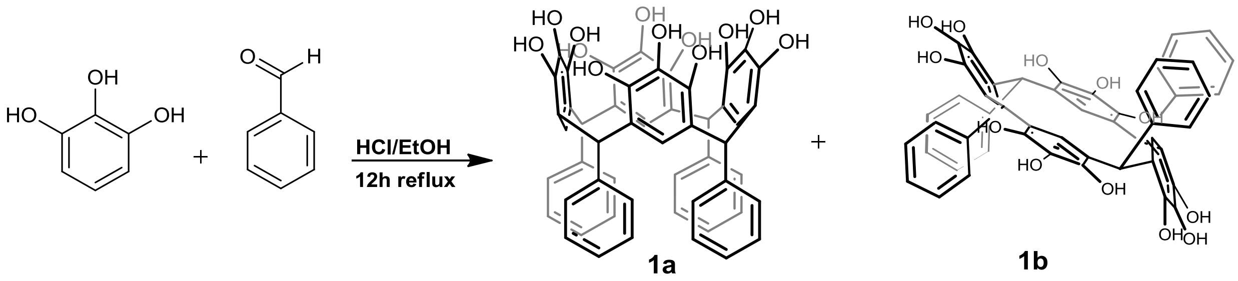

Scheme S1. Synthesis of *C*-tetra(phenyl)pyrogallol[4]arene

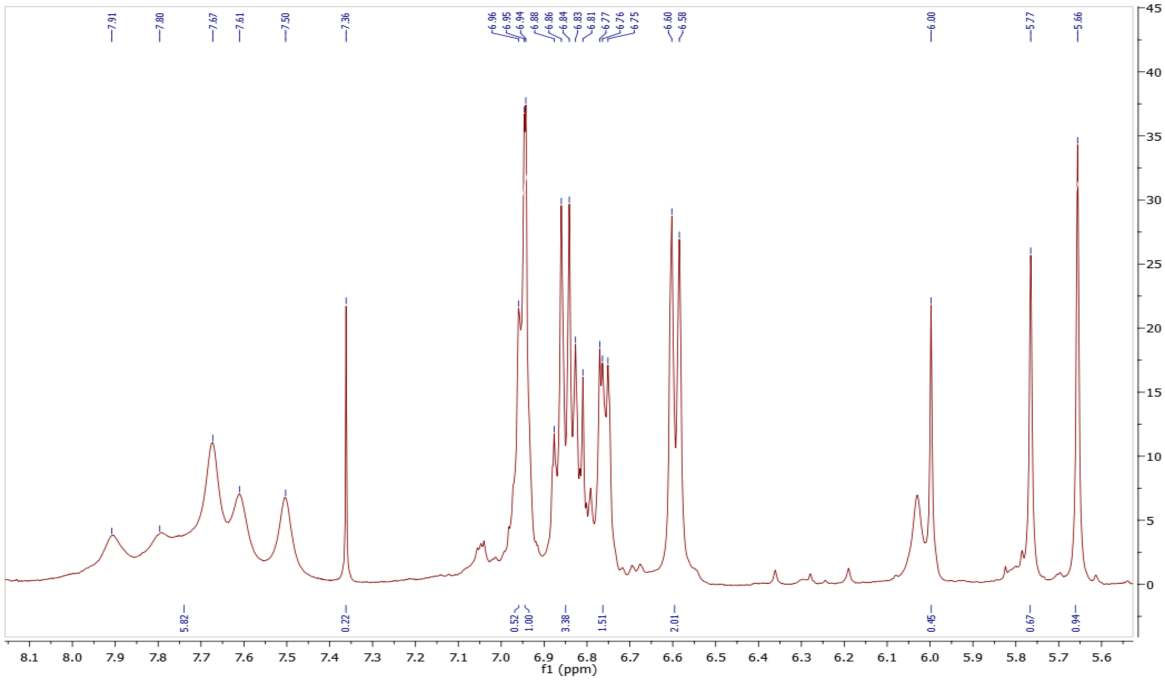

Figure S1. ^{1}H-NMR spectrum - DMSO-d_6 conformational mix (**1a** and **1b)**

1. Crown conformer

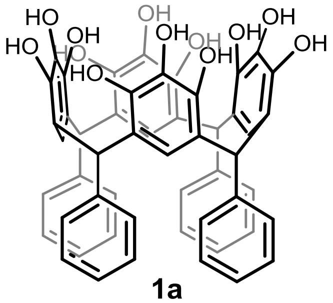

Figure S2. Tetra(phenyl)pyrogallol[4]arene (*crown*)(**1a**)

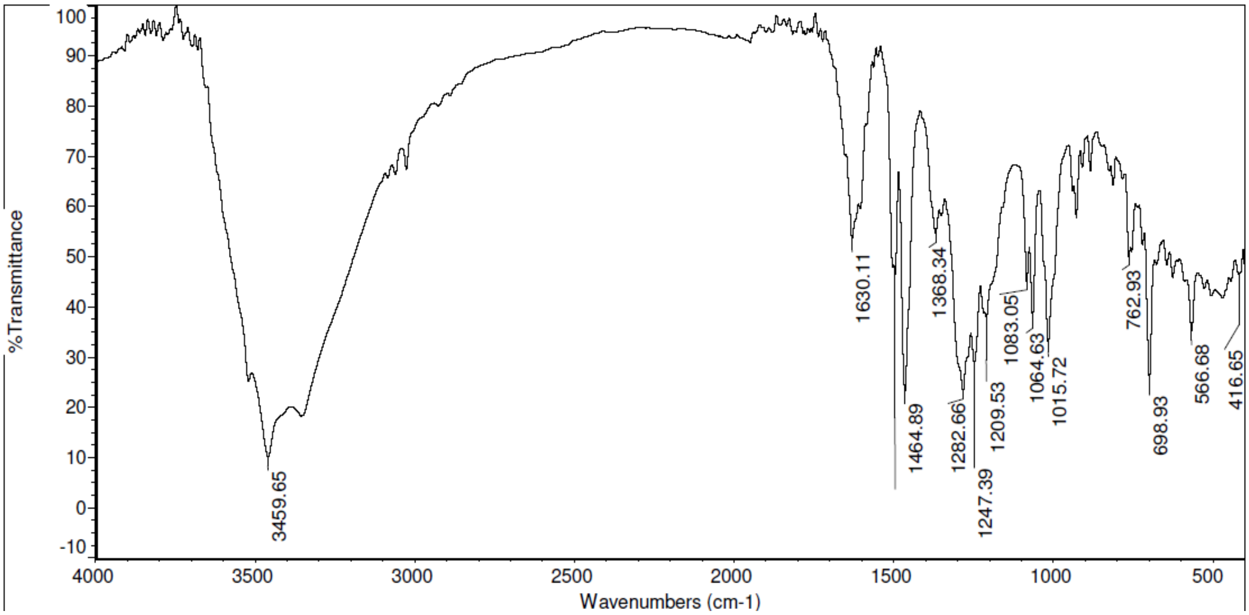

Figure S3. IR Spectrum of compound **1a**

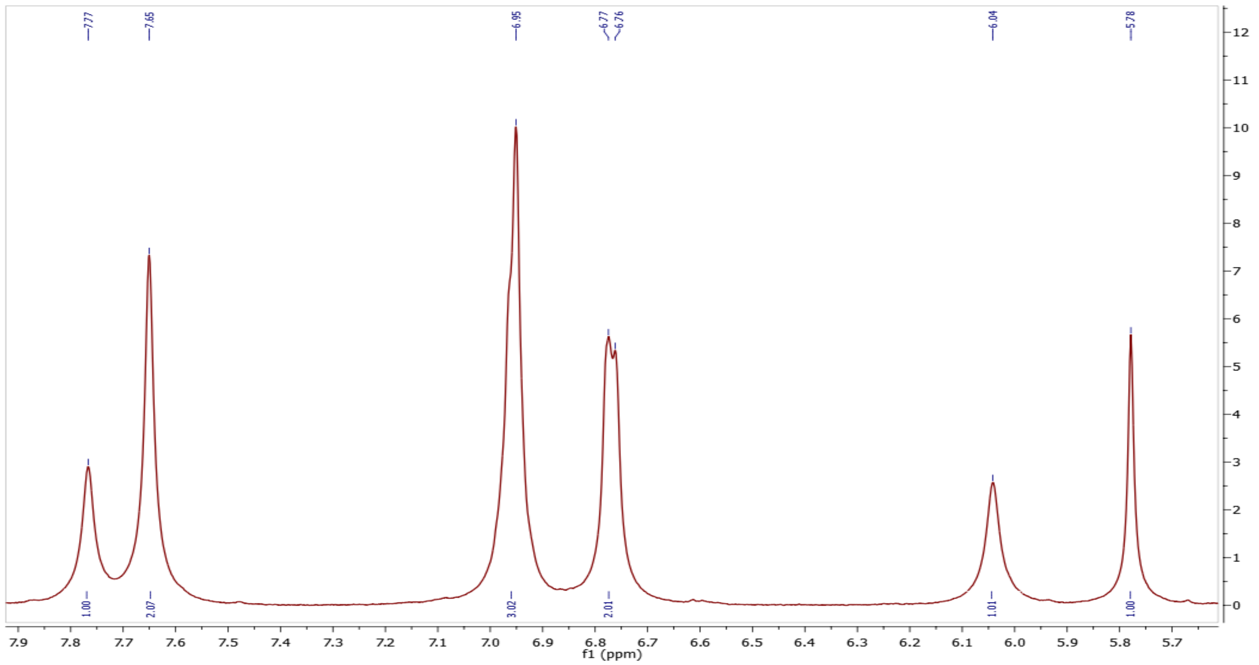

Figure S4. ^{1}H-NMR spectrum DMSO-d_6 of boat conformer**(1a)**

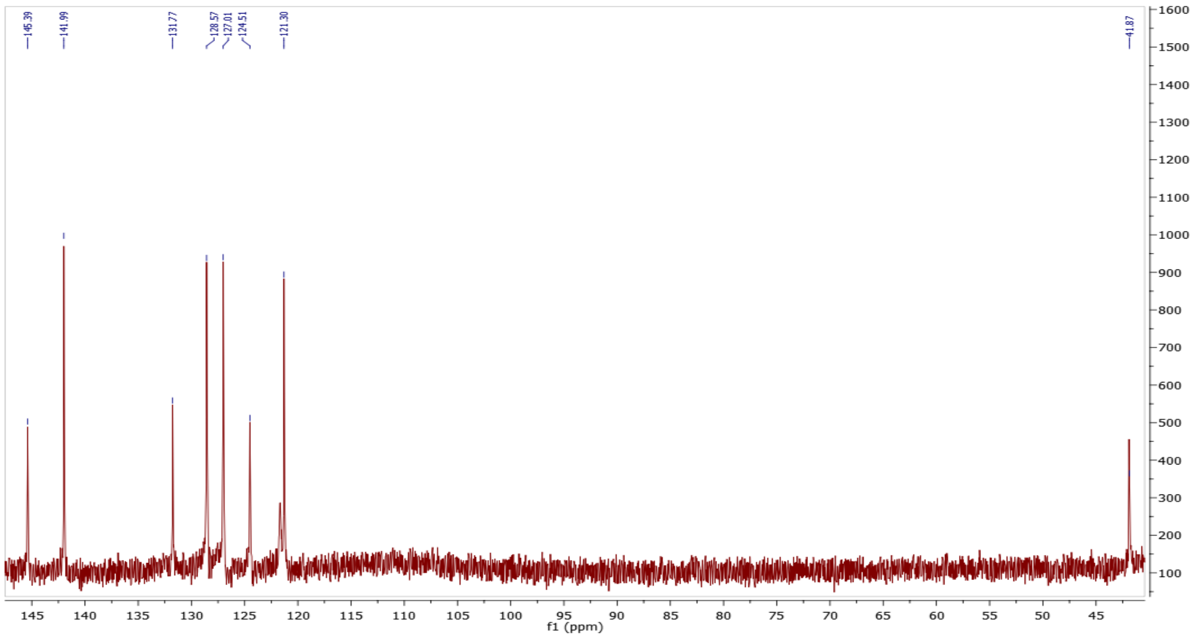

Figure S5. Spectrum ^{13}C-NMR -DMSO-d_6 boat conformer**(1a)**

2. Chair conformer

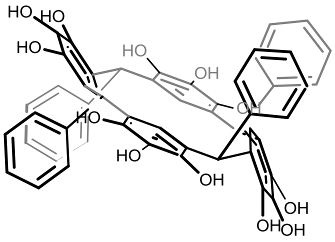

1b

Figure S6. Tetra(phenyl)pyrogallo[4]larene (*chair*)(**1b**)

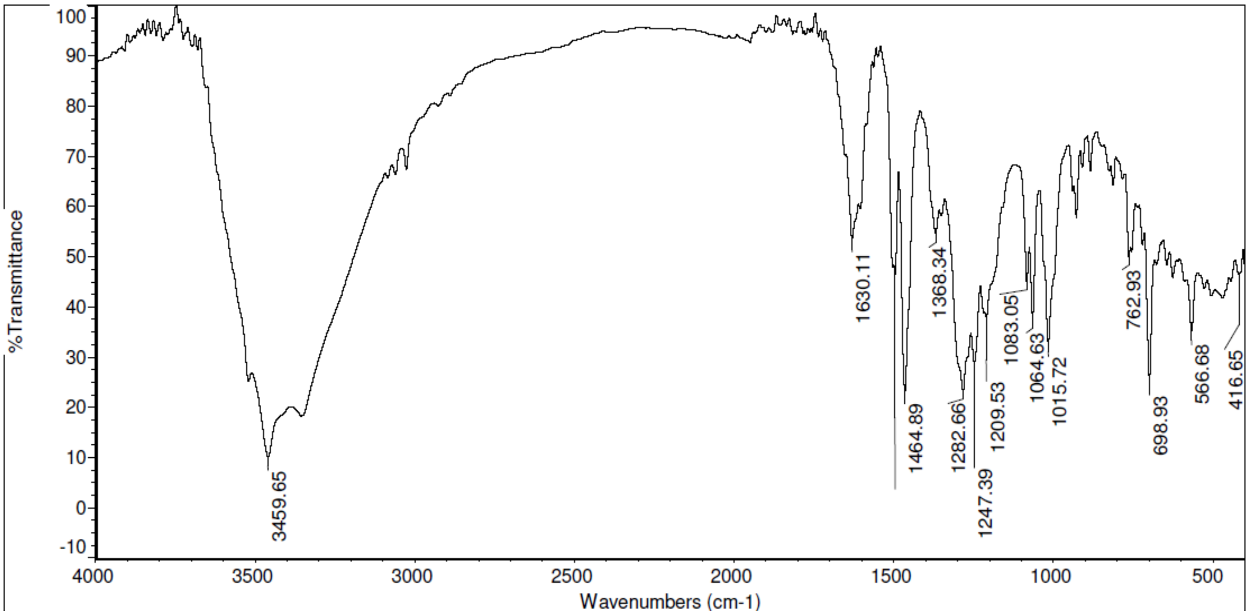

Figure S7. IR Spectrum of compound **1b**

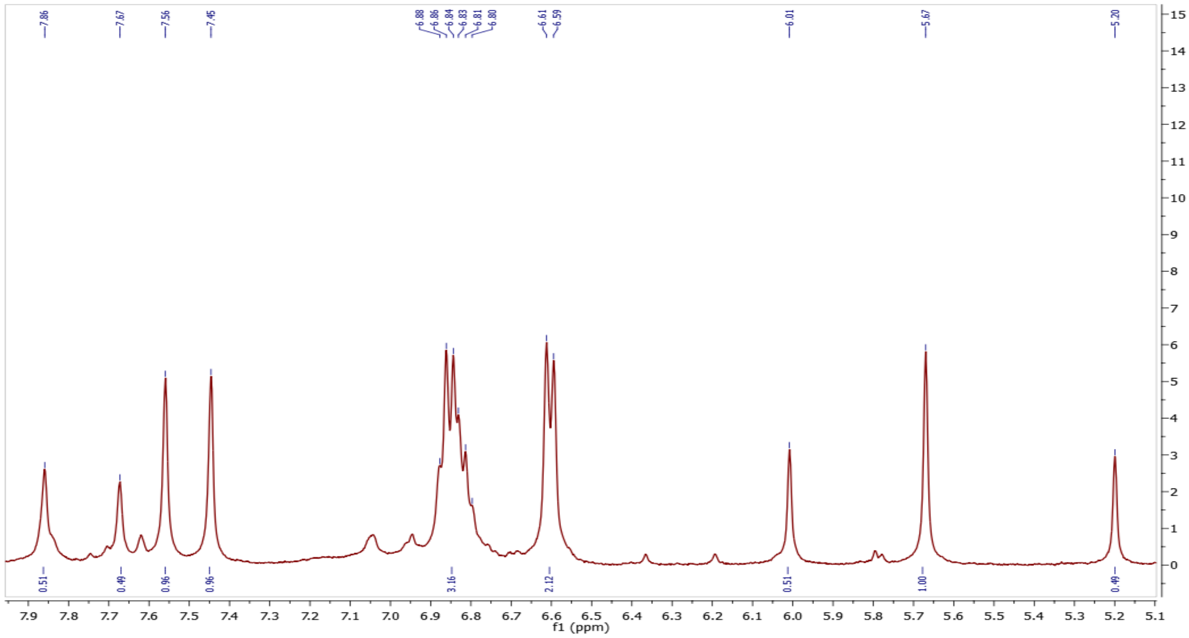

Figure S8. ^{1}H-NMR of chair conformer(**1b**)

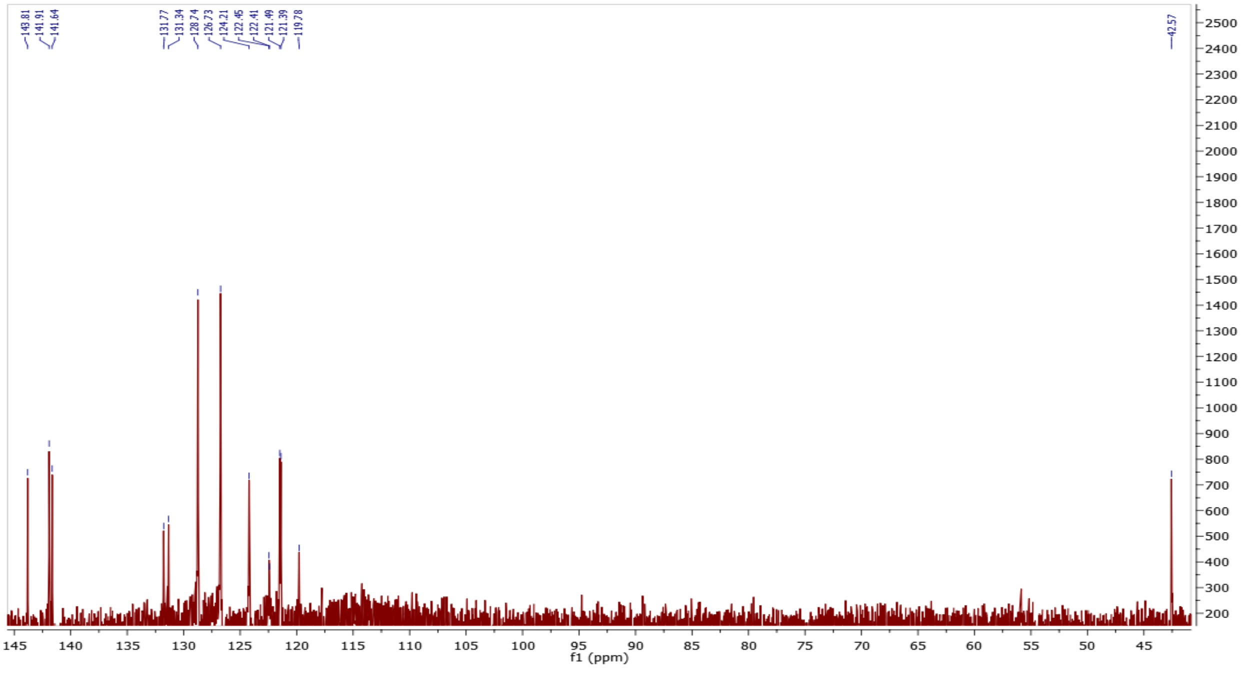

Figure S9. ^{13}C-NMR of chair conformer(**1b**)

Synthesis of the acylated phenylpyrogallol[4]arene

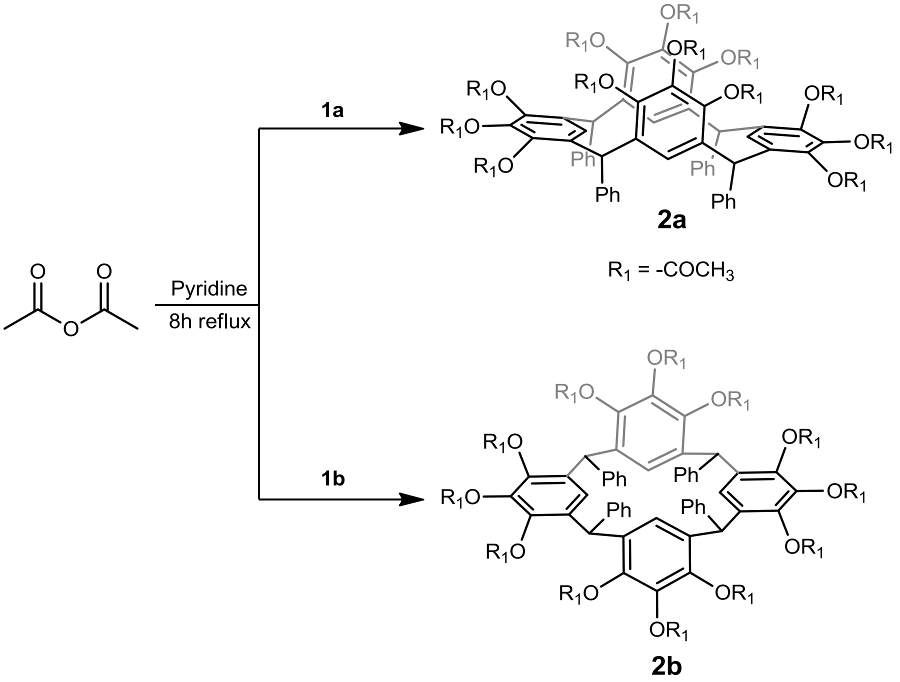

Scheme S2. Synthesis of *C*-tetraphenylpyrogallol[4]arene acetylated (**2a** and **2b**)

1. Boat conformer

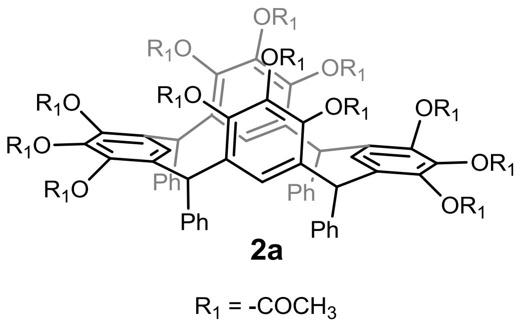

Figure S10. Dodecaacethyl-tetra(phenyl)pyrogallol[4]arene (*Boat*)(**2a**)

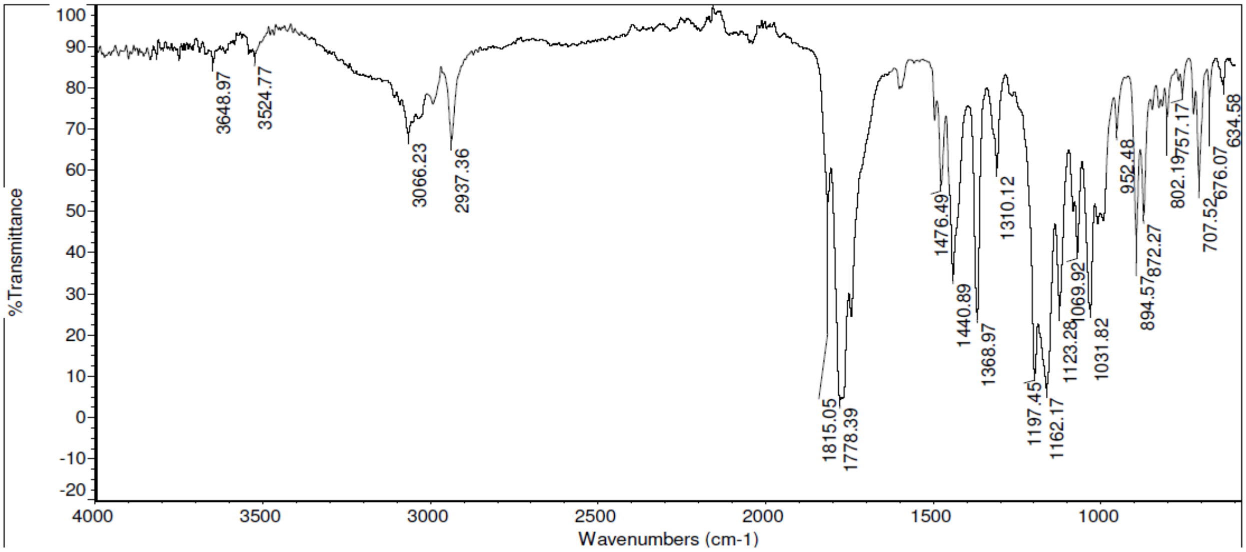

Figure S11. IR spectrum of compound **2a**

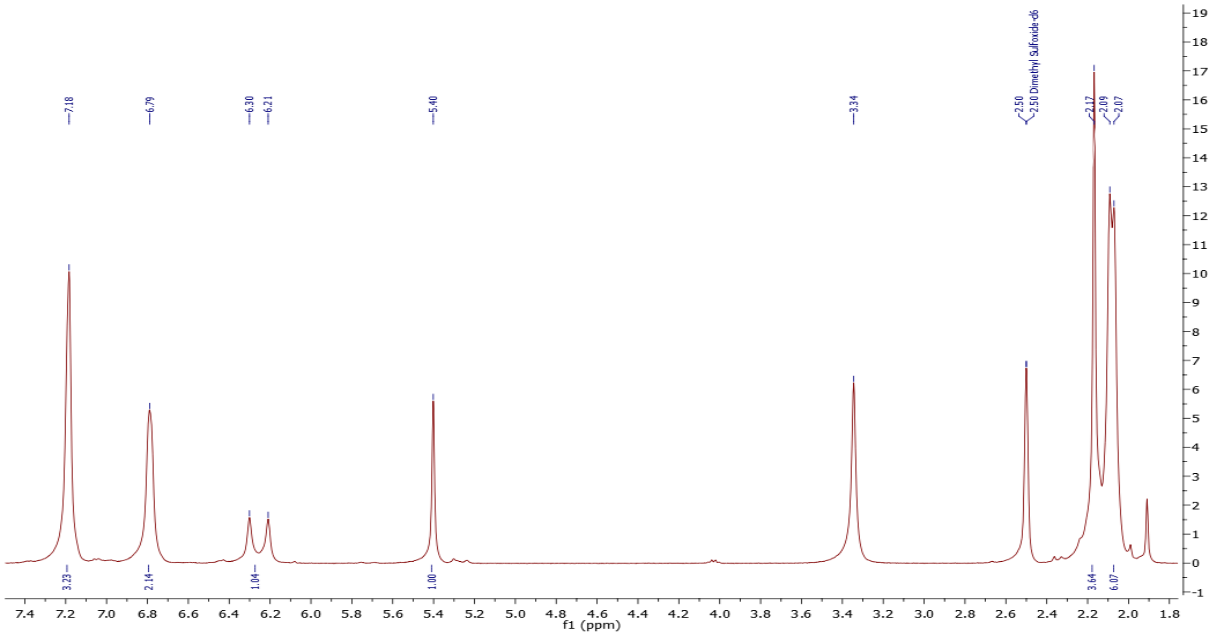

Figure S12. ^{1}H-NMR spectrum boat isomer (**2a)**

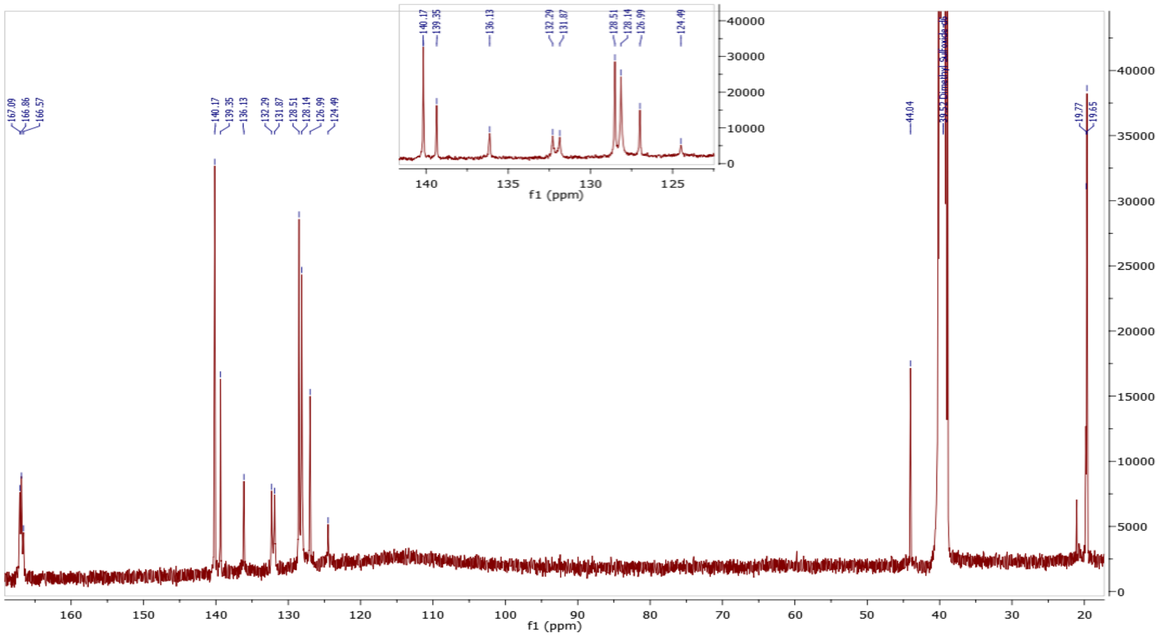

Figure S13. ^{13}C-NMR boat conformer(**2a**)

2. Chair isomer

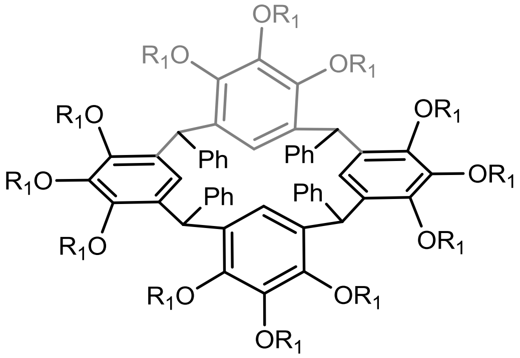

2b

R_1 = -$COCH_3$

Figure S14. Dodecaacethyl-tetra(phenyl)pyrogallol[4]arene (*chair*) (**2b**)

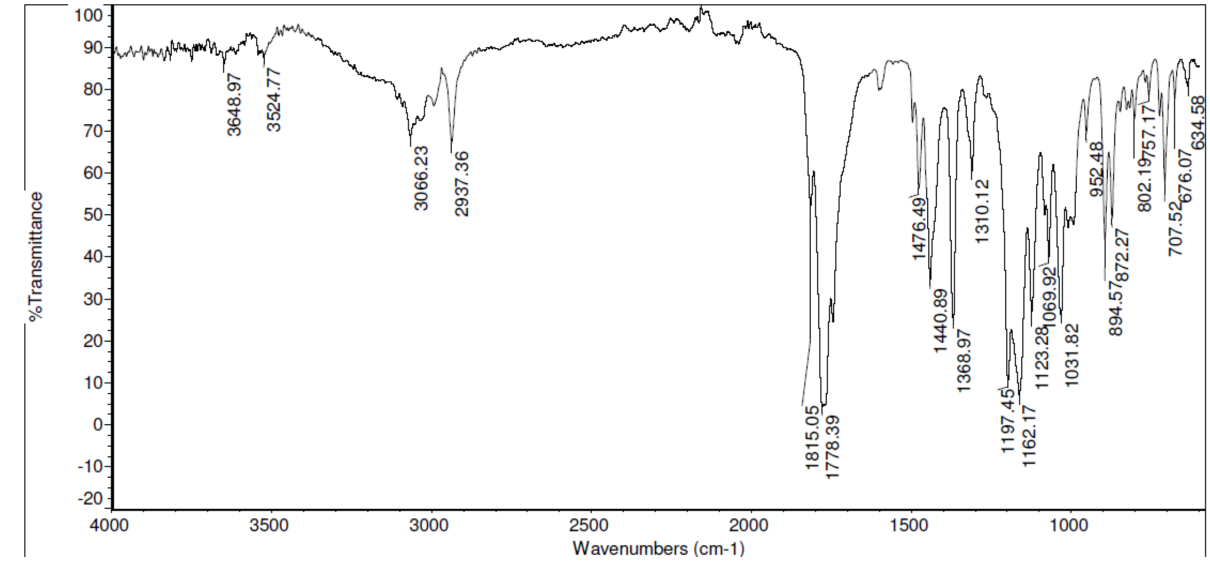

Figure S15. IR of compound **2b**

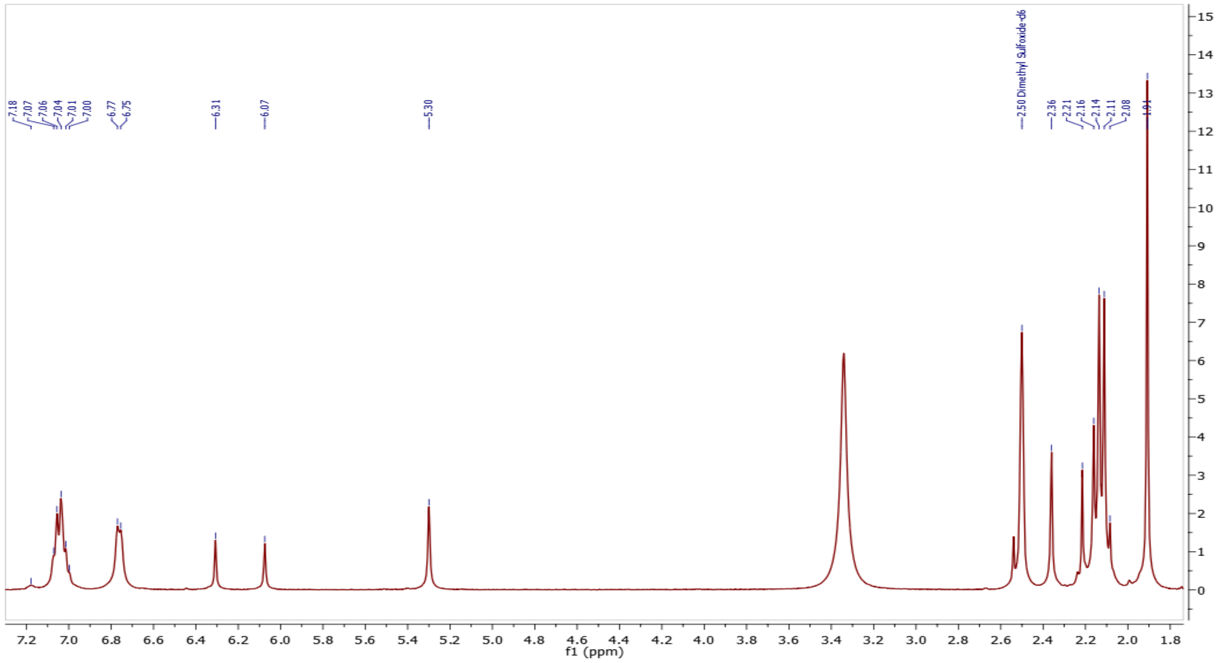

Figure S16. ^{1}H-NMR spectrum of chair isomer(**2b**)

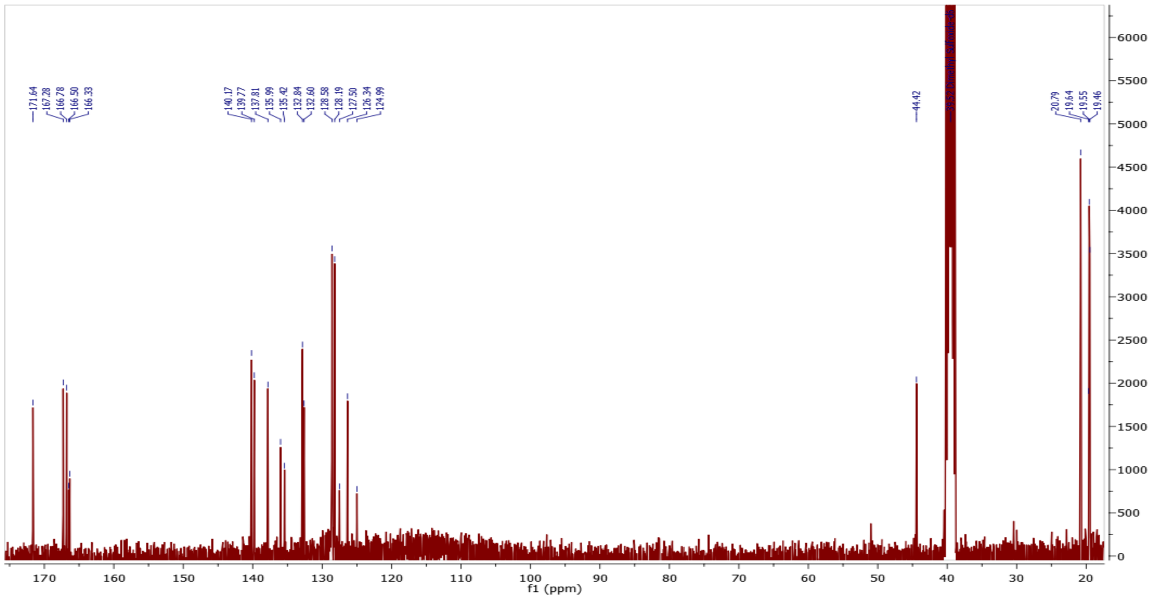

Figure S17. ^{13}C–NMR spectrum of chair isomer(**2b**)
